# P01-10 Workplace Health Promotion To Facilitate Physical Activity Among Swedish Office Workers

**DOI:** 10.1093/eurpub/ckac095.010

**Published:** 2022-08-29

**Authors:** Oskar Ullberg

**Affiliations:** Mälardalens Högskola, Västerås, Sweden

**Keywords:** workplace health promotion, managers, employee, work-life balance, employee branding

## Abstract

**Background:**

The Swedish Work Environmental Authority (2015) states that about 60% of the Swedish workforce work in various office settings, a primarily sedentary environment (Prince, Elliott, Scott, Visintini, Reed, 2019). Today many workplaces offer their employees tax deductible preventive health services, i.e., wellness allowance to promote physical activity. Previous surveys indicate that 50% of the Swedish working population have access to wellness allowance (Hanson, 2007; Weightwatchers, 2017). However, 40% do not take advantage of this benefit (Weightwatchers, 2017; Kjellman & Höglind, 2006). Further, there is still little knowledge about how companies in Sweden promote an active lifestyle at work. This study describes how companies current Workplace Health Promotion (WHP), i.e., wellness allowance and other services related to physical activity, are implemented. Their purpose for providing WHP and the policymakers visions and possible hinders.

**Method:**

Qualitative semi-structured interviews were used to explore how nine policymakers (i.e., human resource managers or CEO) describe WHP related to physical activity analyzed with the framework method.

**Results:**

Physical activity is facilitated through wellness allowance, flexible working hours, step-challenges, walk and talk meetings, and health education. The companies also use the office setting by providing fitness facilities. Accessibility and convenience are described as important for WHP uptake. The provision of WHP related to physical activity is described as employee-driven, where employee initiatives and work-life balance are essential. The purpose of providing WHP described by the informants was to maintain health and productivity among employees and employee branding. Their visions are to reach a broader range of employees by providing easier access to physical activity and more life-friendly solutions. Hinders related to WHP are economic interest, lack of flexibility, and distrust among employees and leaders. The wellness allowance must also be up to date with market prices to be attractive among employees.

**Conclusions:**

Companies have several strategies to facilitate physical activity during and after working hours to maintain employee health and attract and retain top talents. To increase WHP usage and physical activity during and after working hours, we suggest improvements in organizational support, wellness allowance in line with market prices, and easy access to in-house fitness facilities.

